# Association between the serum concentration of triiodothyronine with components of metabolic syndrome, cardiovascular risk, and diet in euthyroid post-menopausal women without and with metabolic syndrome

**DOI:** 10.1186/2193-1801-3-266

**Published:** 2014-05-24

**Authors:** Fabiola Luna-Vazquez, Rosalía Cruz-Lumbreras, Julia Rodríguez-Castelán, Margarita Cervantes-Rodríguez, Jorge Rodríguez-Antolín, Omar Arroyo-Helguera, Francisco Castelán, Margarita Martínez-Gómez, Estela Cuevas

**Affiliations:** Maestría en Ciencias Biológicas, Universidad Autónoma de Tlaxcala, Tlaxcala, México; Doctorado en Ciencias Naturales, Universidad Autónoma de Tlaxcala, Tlaxcala, México; Doctorado en Neuroetología, Universidad Veracruzana, Xalapa, México; Centro Tlaxcala de Biología de la Conducta, Universidad Autónoma de Tlaxcala, Carretera Tlaxcala-Puebla Km 1.5., Tlaxcala, C.P. 90070 México; Instituto de Salud Pública, Universidad Veracruzana, Xalapa, México; Instituto de Investigaciones Biomédicas, Universidad Nacional Autónoma de México, Coyoacán, México

**Keywords:** Metabolic syndrome, Cardiovascular risk, Calorie intake, Thyrotropin, Thyroid hormone, Hyperglycemia

## Abstract

**Purpose:**

To determine the association between the serum concentration of triiodothyronine (T3) with components of metabolic syndrome (MetS), cardiovascular risk (CVR), and diet in euthyroid post-menopausal women without and with MetS.

**Methods:**

A cross-sectional study was performed in 120 voluntary women of an indigenous population from Tlaxcala-México. Euthyroid status was assessed measuring the serum concentration of thyrotropin (TSH) and thyroid hormones, while that of estradiol was measured to confirm the postmenopausal condition. MetS was diagnosed using the American Heart Association/National Heart, Lung, and Blood Institute Scientific Statement (AHA/NHLBI) criterion. Estimation of CVR was calculated based on the Framingham scale. Diet components were evaluated based on survey applications. Correlations, logistic regression analyses, ANOVA or Kruskall-Wallis, and chi-square tests were used to determine significant differences (P ≤ 0.05) between women without MetS and women with MetS having different serum concentrations of T3.

**Results:**

Triiodothyronine was positively correlated with insulin but negatively correlated with glucose, high-density lipoprotein cholesterol (HDL-C), and CVR. Compared to women without MetS, women with MetS and low-normal T3 concentration showed a high risk for hyperglycemia and moderate/high risk for CVR. In contrast, a high-normal T3 concentration increased the risk to have a big waist circumference, a high concentration of HDL-C, and insulin resistance. Diet analysis showed a high grade of malnutrition in women from all groups. The intake of calories was positively affected by the T3 concentration, albeit it did not affect the extent of malnutrition.

**Conclusions:**

In contrast to concentrations of TSH, total thyroxin (T4), and free T4, the concentration of serum T3 was strongly correlated with cardio-metabolic variables in euthyroid postmenopausal women. In comparison to women without MetS, a high-normal serum concentration of T3 in women with MetS is positively associated with reduced glycaemia and CVR but negatively related to body mass index (BMI), insulin, insulin resistance, and HDL-C. Although the analyzed population had a nutritional deficiency, both calories and iron intake were positively affected by the T3 concentration. Our results suggest the necessity of health programs monitoring T3 in old people in order to treat hyperglycemia, cardio-metabolic components, and the ageing anorexia.

## Background

Hypothyroidism (overt or subclinical) has been associated with components of metabolic syndrome (MetS) and cardiovascular diseases (Ichiki 2010). Even in a euthyroid status, a positive correlation between the concentration of free thyroxin (T4) and thyrotropin (TSH) and cardio-metabolic variables has been reported (Roos et al. 2007; Park et al. 2009; Takamura et al. 2009; Chin et al. 2014). Although scarcely studied, the influence of triiodothyronine (T3; free or total) seems to be negative for obesity, insulin concentration, insulin resistance, systolic and diastolic blood pressure, and dyslipidemias (De Pergola et al. 2010; Chin et al. 2014; Roef et al. 2014), but is favored by hyperglycemia (De Pergola et al. 2010).

Some studies have found that the relationship between thyroid hormones and cardio-metabolic variables may be intensified in patients with diabetes, obesity, or MetS (De Pergola et al. 2010; Lambadiari et al. 2011; Taneichi et al. 2011; Tarcin et al. 2012; Topsakal et al. 2012), suggesting that the onset of these pathologies could affect the thyroid function. In agreement, it has been suggested that obese people with a normal thyroid gland may have a hyperactivated hypothalamic-pituitary-thyroid axis, increasing the concentration of TSH and thyroid hormones (Laurberg et al. 2012). Other studies have reported that the presence of MetS components in euthyroid subjects can vary between the lowest and highest serum concentrations of TSH or T4. Thus, the prevalence of MetS increases as the normal-TSH quartile (Park et al. 2009), the risk for insulin resistance increases in the lowest-normal concentration of freeT4 (Roos et al. 2007), and the prevalence of MetS is low at higher free T4 quintiles (Kim et al. 2009). Remarkably, an exploration of cardio-metabolic variables according to normal-T3 concentration has not been done.

Although different factors could influence on the relationship between thyroid hormones and cardio-metabolic variables, composition of diet, which is clearly related to MetS and cardiovascular diseases (Skilton et al. 2008; Motamed et al. 2013), remains unexplored. Despite type of diet and intestinal absorption of thyroid hormones in dogs seem to be uncorrelated (Iemura et al. 2013), the concentration of thyroid hormones is indeed affected by the type of diet (Rabolli and Martin 1977; Serog et al. 1982; Hennemann et al. 1988; Roti et al. 2000). Another important factor to be considered for this relationship is the age. Ageing increases the onset of cardio-metabolic pathologies (Regitz-Zagrosek et al. 2006; Heidari et al. 2010) in response to a nutritional deficiency induced by the ageing anorexia and the loss of dentition (Kmieć et al. 2013; Soenen and Chapman 2013). Ageing also promotes a natural decrease in the pituitary TSH secretion and deiodination of T4, while increases the occurrence of anti-thyroglobulin and anti-thyroperoxidase antibodies (Benseñor et al. 2012). Both quality of diet and ageing could affect the association between thyroid hormones and cardio-metabolic variables. In old subjects, but not in young ones, retinol blood concentration is negatively associated with free T4 and TSH, while zinc is positively associated with free T3 (Ravaglia et al. 2000). Furthermore, the concentration of gastrointestinal molecules involved in the appetite regulation and the ageing anorexia is affected by the thyroid status (i.e. ghrelin and obestatin) (Emami et al. 2014).

The aim of the present study is therefore to determine the association between serum concentration of T3 with components of MetS, CVR, and diet in euthyroid post-menopausal women with and without MetS. An interesting group to do this is the Otomi indigenous population living in Ixtenco-Tlaxcala, México, in which a high prevalence of MetS components has been previously reported (Cruz-Lumbreras et al. 2012).

## Results

Correlation analyses between TSH or thyroid hormones and cardio-metabolic variables were done in euthyroid post-menopausal women. There was no correlation between the serum concentration of TSH and cardio-metabolic variables. Total T4 was negatively correlated with age but positively correlated with diastolic pressure. Free T4 was positively correlated with glucose and diastolic pressure. Total T3 was positively correlated with the body mass index (BMI), the concentration of serum insulin, and the insulin resistance index (HOMA-IR) but negatively correlated with age, serum glucose, and high-density lipoprotein cholesterol (HDL-C). Serum iodine concentration was negatively associated with TSH, but positively correlated with free T4 and T3. Hormone concentrations were not correlated with menarche, menopause age, or the post-menopausal years (Table 1).

**Table 1:** **Correlation analysis between TSH or thyroid hormones and cardio-metabolic variables in euthyroid post-menopausal women**

***Variable***	***TSH***	***T4***	***Free T4***	***T3***
Age (years)	-0.061	-0.205*	-0.036	-0.205*
BMI (kg/m^2^)	-0.108	0.114	0.136	0.220*
Glucose (mg/dL)	-0.023	-0.010	0.261*	-0.264**
Insulin (μUI/mL)	-0.099	0.046	0.030	0.425***
HOMA-IR	-0.079	0.080	0.167	0.239**
Triglycerides (mg/dL)	0.063	0.021	0.011	-0.052
Cholesterol (mg/dL)	-0.045	-0.096	-0.022	-0.100
HDL-C (mg/dL)	0.113	0.034	0.084	-0.266**
Systolic pressure (mmHg)	-0.038	0.067	0.136	-0.072
Diastolic pressure (mmHg)	0.125	0.201*	0.187*	-0.002
Serum iodine (μg/L)	-0.548***	0.008	0.343*	0.286*
Menarche (years)	-0.055	-0.004	-0.014	0.084
Age of menopause (years)	0.035	-0.109	-0.040	-0.157
Years post-menopause	-0.021	-0.129	-0.010	-0.131

TSH, thyrotropin; T4, thyroxine; T3, triiodothyronine. BMI, body mass index; HOMA-IR, insulin resistance; high-density lipoprotein cholesterol (HDL-C). All participants (n = 120) were included for these analyses, except to the serum iodine in which only samples from 52 women were measured. Simple Pearson or Spearman correlation tests were applied. Values are correlation coefficients. *P ≤ 0.05, **P < 0.01, and ***P < 0.001.

Due to the high correlation between T3 and cardio-metabolic variables, women were grouped in: 1) women without MetS (n = 33; without-MetS group), 2) women with MetS and a low T3 concentration (quartile 1; n = 22; group MetS-T3Q1); 3) women with MetS and a low-to-intermediate T3 concentration (quartile 2; n = 21; group MetS-T3Q2); 4) women with MetS and an intermediate-to-high T3 concentration (quartile 3; n = 21; group MetS-T3Q3); and 5) women with MetS and a high T3 concentration (quartile 4; n = 23; group MetS-T4Q4). The serum concentration of TSH was similar between groups and the same was true for that of free T4. The serum concentration of T4 for the MetS-T3Q1 group was significantly lower than that of MetS-T3Q3 and MetS-T4Q4 groups. For its part, the concentration of T3 was low for the MetS-T3Q1 group and high for both MetS-T3Q3 and MetS-T4Q4 groups (Table 2). As far as cardio-metabolic variables are concerned, it was found that: a) age, cholesterol, and blood pressure were similar between groups; b) high values of BMI, insulin, HOMA IR, and HDL-C were found in groups with a high T3 concentration; and c) a high glycaemia was present in groups with low or low-intermediate T3 concentration (Table 2).

**Table 2:** **Mean ± SD of thyrotropin (TSH), thyroid hormones, serum iodine, and cardio-metabolic variables in euthyroid post-menopausal women grouped according to the presence of metabolic syndrome (MetS) and triiodothyronine (T3) concentration**

	Without MetS	MetS-T3Q1	MetS-T3Q2	MetS-T3Q3	MetS-T3Q4	Statistic K or F; P
	n = 33	n = 22	n = 21	n =21	n = 23	
TSH (μUI/mL)	2.8 ± 2.1^a^	2.5 ± 1.4^a^	2.3 ± 1.5^a^	2.6 ± 1.9^a^	2.4 ± 1.8^a^	Ns
T4 (μg/mL)	8.9 ± 0.4^ab^	8.2 ± 1.1^b^	8.4 ± 1.5^ab^	9.7 ± 1.1^a^	9.4 ± 1.2^a^	4.0; 0.002
Free T4 (μg/mL)	1.1 ± 0.2	1.1 ± 0.1	1.1 ± 0.1	1.1 ± 0.2	1.1 ± 0.1	Ns
T3 (μg/mL)	1.2 ± 0.2^ac^	0.9 ± 0.1^b^	1.1 ± 0.04^a^	1.2 ± 0.06^c^	1.4 ± 0.1^d^	62.9; <0.0001
Range of T3 concentration (μg/mL)	(0.81-1.47)	(0.66-0.99)	(0.10-1.13)	(1.14-1.33)	(1.34-1.70)	
Age (years)	67.4 ± 11.8	70.8 ± 8.6	68.9 ± 10.4	66.3 ± 7.5	64.9 ± 10.5	Ns
BMI (kg/m^2^)	25.1 ± 5.3^a^	24.4 ± 3.8^a^	26.6 ± 3.5^ab^	26.8 ± 3.0^ab^	28.6 ± 5.0^b^	14.9; 0.004
Glucose (mg/dL)	100.6 ± 30.6^a^	133.7 ± 47.5^bc^	158.0 ± 58.9^c^	135.5 ± 56.0^bc^	109.7 ± 57.5^ab^	28.5; <0.0001
Insulin (μUI/mL)	9.2 ± 5.8^a^	8.8 ± 8.6^a^	10.0 ± 6.5^a^	12.6 ± 6.6^ab^	16.4 ± 7.0^b^	27.1; <0.0001
HOMA-IR	2.2 ± 1.4^a^	3.1 ± 3.4^ab^	3.6 ± 1.8^abc^	4.3 ± 3.0^bc^	4.4 ± 2.0^c^	25.5; <0.0001
HDL-C (mg/dL)	51.0 ± 19.3^a^	44.4 ± 9.6^ab^	48.7 ± 30.0^ab^	40.1 ± 6.0^b^	39.7 ± 10.0^ab^	12.0; 0.01
Cholesterol (mg/dL)	198.5 ± 6.2	215.5 ± 13.6	197.9 ± 8.5	183.8 ± 9.3	195.6 ± 7.7	Ns
Systolic pressure (mmHg)	118.2 ± 3.1^a^	125.5 ± 3.2^ab^	133.3 ± 3.9^b^	124.3 ± 2.9^ab^	129.6 ± 2.9^ab^	12.3; 0.01
Diastolic pressure (mmHg)	73.6 ± 1.6	73.1 ± 1.5	73.3 ± 2.4	74.7 ± 2.4	75.7 ± 1.8	Ns

T4, thyroxine; BMI, body mass index; HOMA-IR, insulin resistance index; high-density lipoprotein cholesterol (HDL-C). ANOVA or Kruskal-Wallis tests and Tukey-Kramer or Dunns post-hoc test were applied. Ns: no significant. Different letters indicate statistical differences between groups reported by post-hoc tests.

Comparing to women without and with MetS, binomial logistic regression analyses showed that the risk to have MetS components varies according to the T3 serum concentration. For waist circumference and HOMA-IR, the odd ratio (OR) was high for MetS-T3 (Q2-Q4) groups, while for the HDL-C the odd ratio was high for MetS-T3Q3 and Q4 groups. The odd ratio to have an elevated arterial tension and serum triglycerides was higher in women with MetS that those without MetS, regardless of T3 concentration. Women with MetS and a low concentration of T3 had a high odd ratio for hyperglycemia and CVR than those having a high T3 concentration (Table 3).

**Table 3:** **Percentage and odds ratios (CI 95%) for metabolic syndrome (MetS) components and cardiovascular risk (CVR) in euthyroid post-menopausal women grouped considering the presence of MetS and triiodothyronine (T3) concentration**

	Without MetS n=33	MetS-T3Q1 n=22	MetS-T3Q2 n=21	MetS-T3Q3 n=21	MetS-T3Q4 n=23
Waist circumference	30.3%	45.5%	66.7%	85.7%	82.6%
	REF	2.8 (0.8-9.7)	6.4 (1.7-23.5)**	16.2 (3.6-72.8)***	12.7 (3.0-53.3)***
Arterial tension	30.3%	72.7%	81.0%	61.9%	69.5%
	REF	5.7 (1.6-19.2)**	10.0 (2.6-38.6)***	4.1 (1.2-13.3)*	6.2 (1.8-21.0)**
Hyperglycemia	27.3%	95.5%	85.7%	66.7%	65.2%
	REF	53.8 (6.2-462.2)***	15.8 (3.7-67.4)***	5.4 (1.6-18.1)**	5.2 (1.6-16.9)**
Triglycerides	24.2%	72.7%	52.4%	57.1%	65.2%
	REF	8.0 (2.3-27.8)**	3.3 (1.0-10.9)*	4.2 (1.3-13.7)*	6.0 (1.8-19.6)**
Low HDL-C	54.5%	76.2%^(21)^	76.2%	95.2%	85.7% ^(21)^
	REF	2.8 (0.8-9.5)	2.7 (0.8-9.2)	16.4 (1.9-137.6)*	4.9 (1.2-20.0)*
HOMA-IR	24.2%	36.3%	66.7%	66.7%	78.2%
	REF	2.4 (0.6-8.5)	8.3 (2.2-30.5)***	6.8 (1.9-24.2)**	12.3 (3.2-47.1)***
CVR moderate/high	6.1%	45.5%^(21)^	52.4%	47.6%	19.0%^(21)^
	REF	14.6 (2.7-78.6)***	17.4 (3.2-92.9)***	14.0 (2.6-74.3)**	3.5 (0.5-21.7)

Binomial logistic regression was applied adjusting age was for all cases. HDL-C, HDL cholesterol; HOMA-IR, insulin resistance. *P ≤ 0.05, **P < 0.01, and ***P < 0.001. Note: HDL-C was not possible to be measure for all women. Numbers into superscript parenthesis together to prevalence for groups MetS-T3Q1 and MetS-T3Q4 indicate the number of participants for HDL-C and CVR.

All groups had a low calorie intake that was accompanied by a diminished intake of fiber, calcium, selenium, ascorbic acid, vitamin E, and folate (Table 4). In highly contrast, all women showed a high intake of iron (Table 4). Both the intake of calories and iron were positively affected by T3 concentration. Percentages for other dietetic alterations were similar between groups (Table 4).

**Table 4:** **Means ± SD of dietetic variables, percentage of dietetic deficiencies, and odds ratio (OR) of dietetic alterations for euthyroid post-menopausal women considering the presence of metabolic syndrome (MetS) and triiodothyronine (T3) concentrations**

	Without MetS n=29	MetS-T3Q1 n=20	MetS-T3Q2 n=16	MetS-T3Q3 n=18	MetS-T3Q4 n=19	Statistic K or F; P; χ2; P
Caloric intake (kcal/day)	1401.1 ± 514.3	1217.8 ± 380.1	1301.2 ± 500.3	1310.4 ±300.1	1584.7 ± 598.7	Ns
Low calories intake <1600 Kcal/day	69%^a^	95%^b^	68.8%^ab^	83.3%^ab^	52.6%^a^	10.3; 0.03
OR (CI 95%) for low calories intake without age adjusted	REF	8.5 (0.9-74.0)*	0.9 (0.2-3.6)	2.2 (0.5-9.7)	0.5 (0.1-1.6)	
OR (CI 95%) for low calories intake^ǂ^	REF	7.3 (0.8-65.8)^┼^	1.0 (0.2-4.0)	2.3 (0.5-10.6)	0.5 (0.1-2.0)	
Protein intake (g/day)	50.8 ± 20.4	46.8 ± 16.0	51.7 ± 19.7	49.4 ± 16.6	63.7 ± 28.7	Ns
Low protein intake (range 10-35%)	6.9%	5%	12.5%	5.5%	0.0%	Ns
OR (CI 95%) for low protein intake^ǂ^	REF	0.6 (0.05-7.9)	1.9 (0.2-16.1)	0.7 (0.6-9.7)	NA	
Lipid intake (g/day)	36.31 ± 24.9	31.6 ± 17.9	30.4 ± 15.8	34.8 ± 15.6	42.6 ± 27.7	Ns
Low lipid intake (range 20-35%)	48.3%	35.0%	43.8%	22.2%	31.5%	Ns
OR (CI 95%) for low lipid intake^ǂ^	REF	0.5 (0.1-1.8)	1.0 (0.2-3.6)	0.3 (0.09-1.4)	0.8 (0.2-3.1)	
Carbohydrate intake (g/day)	218.4 ± 71.5	186.6 ± 64.1	205.3 ± 101.5	200.4 ± 49.6	236.7 ± 74.4	Ns
High carbohydrate intake (range 45-65%)	44.8%	25.0%	25.0%	22.2%	26.3%	Ns
OR (CI 95%) for high carbohydrate intake^ǂ^	REF	0.4 (0.1-1.4)	0.4 (0.1-1.8)	0.4 (0.1-1.5)	0.6 (0.1-2.4)	
Fiber (g/day)	15.2 ± 5.6	14.2 ± 6.6	15.0 ± 4.2	14.5 ± 3.3	18.7 ± 5.9 6	Ns
Low fiber intake <22 g/day	86.2%	85.0%	93.8%	100%	8.4%	Ns
Calcium (mg/day)	991.2 ± 443.9	909.8 ± 333.0	1071.5 ± 488.1	909.9 ± 326.1	1227.6 ± 589.9	Ns
Low calcium intake <1200 mg/day	69.0%	80.0%	68.8%	88.9%	52.6%	Ns
Sodium (mg/day)	1418.5 ± 581.6	1333.1 ± 577.8	1379.8 ± 411.3	1405.5 ± 328.9	1735.4 ± 578.5	Ns
High sodium intake <2300 mg/day	10.3%	10.0%	6.3%	0.0%	15.7%	Ns
Phosphorous (mg/day)	761.3 ± 301.1	680.7 ± 217.0	810.2 ± 432.7	737.4 ± 229.8	885.9 ± 329.4	Ns
Low phosphorous intake <700 mg/day	48.3%	65.0%	37.5%	50.0%	36.0%	Ns
Iron (mg/day)	31.2 ± 10.3^ab^	28.6 ± 12.2^a^	31.1 ± 8.4^ab^	29.3 ± 6.3 ab	38.3 ± 12.5^b^	2.6; 0.03
Low iron intake <8 mg/day	0.0%	0.0%	0.0%	0.0%	0.0%	Ns
Selenium (mg/day)	25.8 ± 23.7	25.2 ± 20.6	33.3 ± 24.3	25.7 ± 19.9	37.6 ± 25.1	Ns
Low selenium intake <55 mg/day	82.8%	95.0%	75.0%	83.3%	63.1%	Ns
Ascorbic acid (mg/day)	30.1 ± 31.7	46.1 ± 72.3	35.2 ± 28.5	28.0 ± 33.5	29.6 ± 13.8	Ns
Low ascorbic acid intake <75 mg/day	89.7%	90.0%	93.8%	94.4%	100.0%	Ns
Vitamin A (μg ER/day)	621.8 ± 476.1	615.7 ± 338.4	752.8 ± 462.2	605.8 ± 324.2	768.4 ± 352.9	Ns
Low vitamin A intake <700 mg/day	62.1%	65.0%	50.0%	61.1%	47.3%	Ns
Vitamin E (mg/day)	4.2 ± 3.3	6.3 ± 4.5	4.9 ± 2.5	6.0 ± 3.9	5.6 ± 3.1	Ns
Low vitamin E intake <15 mg/day	100.0%	95.0%	100.0%	100.0%	100.0%	Ns
Folate (mcg/day)	71.0 ± 76.1	91.0 ± 88.1	66.3 ± 68.8	67.0 ± 79.6	70.5 ± 32.9	Ns
Low folate intake <400 mcg/day	100.0%	100.0%	100.0%	100.0%	100.0%	Ns

Note: Diet survey was applied to 102 women. ANOVA or Kruskal-Wallis tests and Tukey-Kramer or Dunn’s post-hoc tests, and chi-square were applied. ^ǂ^Binomial regression analysis (age and BMI adjusted) were done for macronutrients. Ns: no significant. Different letters indicate statistical differences between groups reported by post-hoc tests. *P = 0.05; ^┼^P = 0.07.

## Discussion

In contrast to concentrations of TSH, total T4, and free T4, the concentration of serum T3 was strongly correlated with cardio-metabolic variables in postmenopausal Otomi women. Although the concentration of T3 seems to be unrelated to the MetS, the presence of their components varies in line with quartiles of the normal-concentration of this hormone. Low-normal values of T3 are related to the presence of a high glucose concentration and a moderate-high CVR. Meanwhile, high-normal values of T3 are associated with a high insulin level, HOMA-IR, a high BMI, and low HDL-C. Triglycerides and arterial pressures did not have a clear relation with the serum concentration of T3. The analysis of diet composition showed an evident malnutrition. Most women enrolled in this study ingest a low content of calories, lipids, minerals, and vitamins. The quality of the diet was not correlated with the presence of MetS, albeit women having a high-normal concentration of T3 consume more calories and iron, which was not enough to improve the extent of malnutrition.

There is much information about the relation between T4 or TSH and cardio-metabolic variables (De Pergola et al. 2010; Lambadiari et al. 2011; Taneichi et al. 2011; Tarcin et al. 2012), but only a few studies have been done in old people, and particularly in post-menopausal women (Park et al. 2009; Takamura et al. 2009; Topsakal et al. 2012). In agreement with our results, a high T3 (total or free) concentration has been associated with a high BMI (De Pergola et al. 2010; Taneichi et al. 2011; Tarcin et al. 2012; Roef et al. 2014), which may be related to the promotion of adipocyte differentiation (Flores-Delgado et al. 1987). Triiodotironine also shows a positive association with insulin resistance (Lambadiari et al. 2011; Tarcin et al. 2012) but a negative association with hyperglycemia (Taneichi et al. 2011). This fact could be explained because the T3 affects the absorption of glucose in the gastrointestinal tract, the liberation of glucose by hepatic tissue, cellular glucose transport, insulin secretion by pancreas, and the glucose utilization by muscle cells (Wang 2013).

Our present results about the positive correlation between T3 and CVR agree with other studies reporting that low T3 levels are present in old women/men with heart failure, coronary artery disease, atherosclerosis, myocardial infarction or cardiomyopathies (Selvaraj et al. 2012; Yang et al. 2012). In contrast, the HDL-C is negatively affected by serum T3 levels although this could not ameliorate the positive actions of T3 on vascular function (Takamura et al. 2009; De Pergola et al. 2010; Chin et al. 2014; Roef et al. 2014). These actions of T3 on the vascular smooth muscle relaxation are well known (Ojamaa et al. 1993), as well as its negative relation with pro-inflammatory molecules (Lubrano et al. 2010).

In the present study, women had a low intake of calories, and an important deficiency of minerals and vitamins. Such malnutrition could be associated with the ageing anorexia and loss of dentition (Kmieć et al. 2013) as well as to the extent of poverty present in indigenous communities (Gracey and King 2009). This finding is important due to the relevance of an adequate nutrition in old people to avoid cardiovascular and metabolic diseases (Da Silva and Rudkowska 2014). In this regard, we previously found a high prevalence of overweight, obesity, type two diabetes mellitus (T2DM), hypertriglyceridemia, and MetS in old Otomi women (Cruz-Lumbreras et al. 2012). Given the present results is tentative to speculate that the latter metabolic alterations are associated with the malnutrition reported in the present study. However, our results could not be compared to other studies because the lack of information about nutrition of older indigenous from México. Some studies have shown that Tarahumara children have a deficient ingest of iodine, zinc, vitamin B12, iron, and folate (Monárrez-Espino et al. 2004; Monárrez-Espino and Greiner 2005), which suggests a similar condition for old people. A particular mention should be done about iron intake in Otomi women, because most of the intake of this mineral corresponds to non-heme iron (from leguminous plants) that is absorbed in less extent (MacPhail 2001).

Although the type of diet was similar between groups, there was a positive association between the serum normal-concentration of T3 with a high caloric intake that could be associated with a high body weight and intake of iron. These results agree with other studies (Serog et al. 1982; Roti et al. 2000) and may be linked to the promotion of appetite by T3 (Emami et al. 2014). However, a high-normal concentration of T3 was not able to relieve the anorexia and the consequent weight loss at ageing (Kmieć et al. 2013). The latter could occur also for mineral and vitamin deficiencies possibly due to the bad quality of the diet ingested by this indigenous group. In this way, a nutritional intervention on the old population could be necessary to avoid cardio-metabolic alterations and to increase thyroid hormones concentrations. It is known that a dietary imbalance is related to MetS (Al-Daghri et al. 2013), and the supplementation of vitamin A increases the serum T3 concentration (Farhangi et al. 2012).

## Conclusions

In contrast to serum concentrations of TSH, total T4, and free T4, the concentration of serum T3 was strongly correlated with cardio-metabolic variables in euthyroid postmenopausal women. In comparison to women without MetS, a high-normal serum concentration of T3 in women with MetS is positively associated with reduced glycaemia and CVR but negatively related to BMI, insulin, HOMA-IR, and HDL-C. Although the analyzed population had nutritional deficiency, both the intake of calories and iron were positively affected by the T3 concentration. Our results suggest the necessity that health programs monitoring T3 in old people in order to treat hyperglycemia, cardio-metabolic components, and the ageing anorexia.

## Methods

This was a cross-sectional study in volunteer post-menopausal indigenous women from Ixtenco-Tlaxcala, México. Post-menopause was defined as the lack of the menstrual period occurring naturally for at least three years (Heidari et al. 2010). Participants were recruited through personal invitations that were done by the local Health Office from Ixtenco-Tlaxcala. Women were physically active, could skillfully walk, and most of them were farmer. The criterion to be considered as euthyroid women was to have a concentration of TSH <10 μUI/mL (Benseñor et al. 2012). Women with chronic liver or renal diseases, cancer, neurological or psychological illness (depression, epilepsy, and schizophrenia), thyroidectomy, and those being under treatment with estrogen, androgen, or lithium medication were excluded. This study was done in accordance with the Helsinki Declaration of Human Studies and was approved by Ethical Committees of the Health Office from the State of Tlaxcala and the Universidad Autónoma de Tlaxcala. All participants provided a written inform consent. Anthropometric and clinical measurements were obtained by specialized personnel. Menarche and menopause age were also recorded. Thus, 120 women having more than 45 years old (corresponding to 13.5% of eligible population from Ixtenco, Tlaxcala) were included in this study.

### Metabolic syndrome determination

Metabolic syndrome was diagnosed using the American Heart Association/National Heart, Lung, and Blood Institute Scientific Statement (AHA/NHLBI) (Guize et al. 2008) criterion that considers: *a*) abdominal obesity, defined as a waist circumference ≥88 cm; *b*) serum triglycerides ≥150 mg/dL or to be under current medication for dyslipidemias; *c*) HDL-C <50 mg/dL; *d*) blood pressure ≥130/85 mmHg or to be under current use of antihypertensive medication; and *e*) fasting plasma glucose ≥100 mg/dL or to be under current use of hypoglycemic medication. Serum concentration of glucose, total cholesterol and triglycerides were measured using standard enzymatic methods (ELITech, France). The HDL-C was measured using a precipitating method (ELITech, France). Blood pressures monitor (Homecare, México) and stethoscope (Medimetrics, México) were used to measure both systolic and diastolic pressures. The BMI was calculated as weight (kg) divided by the square of height (m^2^) and was used as an index of overall adiposity. Due to elderly subjects commonly are shorter and slimmer than their younger counterparts, the height was estimated by the half arm span (Shahar and Pooy 2003).

### Hormone measurements

Blood samples were obtained from ante-cubital veins of participants having a fast of 12 h. Serum was allowed to clot and then separated by centrifugation and stored at -20°C. Serum concentration of TSH, total T4, free T4, total T3, and estradiol were measured using a chemiluminescence method that was carried out by CARPERMOR Laboratories, S.A. de C.V. Manufacturers were blinded to the thyroid history of participants and to any other data from them. Serum concentration of insulin was also measured by chemiluminescence, and insulin resistance was calculated using the HOMA-IR [fasting insulin (μU/mL) x fasting serum glucose (mg/dL)/405], using a cutoff ≥ 2.7 (Topsakal et al. 2012).

### Serum iodine measurement

Serum iodine was measured using a method based on the Sandell–Kolthoff reaction. Briefly, serum proteins were precipitate and afterwards incinerated in an alkaline medium. Iodine was determined by the rate of color disappeared at 504 nm. Iodine bound to protein was reported as μg/L. Sensibility of the assay was 0.005 μg/L. A concentration of serum iodine ranging from 50 to 100 μg/L was considered as normal (Risher and Keith 2009).

### Cardiovascular risk calculation

The CVR was determined using the Framingham scale that considers risk factors such as age, sex, lipids, systolic blood pressure, smoking habits, and glucose levels (Wilson et al. 1998). According to the Expert Committee on the Diagnosis and Classification of Diabetes Mellitus (2003), the T2DM was diagnosed when a fasting glucose concentration ≥ 126 mg/dL was measured or when participants had been previously diagnosed. Hypertension was diagnosed when the systolic pressure was ≥ 140 mmHg; the diastolic pressure was ≥ 90 mmHg or when women had a current use of anti-hypertensive medication (Wilson et al. 1998). A moderate risk was considered for 15-20% of score, and a high risk was >20%.

### Diet analysis

Dietary information was collected by the 24-hours recall method, applying personal interviews from Tuesday to Friday at participant’s address. Carbohydrate, protein, lipid, vitamins, and minerals intake were calculated using the Mexican Food Equivalent System (Perez-Lizaur et al. 2008) and Dietary Guidelines for Americans (2010). Total intake calories were compared with resting metabolic rate estimated by Harris-Benedict formula (Melzer et al. 2007). Nutrient recommendations were based on the Dietary Guidelines for Americans (2010).

### Statistical analysis

Data were analyzed using the Statistical Package for the Social Sciences software version 17. Correlation analyses involving thyroid hormones or TSH and cardio-vascular variables from all participants (unless otherwise is mentioned) were carried out using Pearson or Spearman analyses. To analyze the association of normal-concentration of T3 with cardio-metabolic and dietetic variables, women were allocated in five groups. The control group was constituted by women without MetS, the rest of groups were women with MetS organized by quartiles to T3 (MetS-T3Q1 to MetS-T3Q4). Statistical comparisons between variables were done using Kruskal Wallis, ANOVA analyses, or chi-square tests as it was necessary. Binary logistic regression analysis was used to determine the risk to present any cardio-metabolic (adjusting age) or macro-nutritional alterations (adjusting age and BMI) between groups. Data are expressed as mean ± standard deviation (SD), prevalence (%), or OR and 95% confidence intervals (CI). A statistical significance was considered at a P ≤ 0.05.

## Authors’ information

FLV Nutritionist, Master. RCL Chemist, PhD. JRC Nutritionist, student from a Master. MC Nutritionist, PhD. JRA Chemist, PhD. OAH Biologist, PhD. FC Chemist, PhD. MMG Biologist, PhD. EC Chemist, PhD.

## Abbreviations

AHA/NHLBI: American Heart Association/National Heart, Lung, and Blood Institute Scientific Statement
BMI: Body mass index
CVR: Cardiovascular risk
CI: Confidence interval
HDL-C: High-density lipoprotein cholesterol
HOMA-IR: Insulin resistance index calculated by the Homeostasis Model Assessment
MetS: Metabolic syndrome
OR: Odds ratio
Q: Quartile
SD: Standard deviation
TSH: Thyrotropin
T4: Thyroxin
T2DM: Type 2 diabetes mellitus
T3: Triiodothyronine
VCAM: Vascular adhesion molecule.
